# Genetic Detection and Isolation of *Crimean-Congo hemorrhagic fever virus*, Kosovo, Yugoslavia

**DOI:** 10.3201/eid0808.010448

**Published:** 2002-08

**Authors:** Anna Papa, Bojana Boźović, Vassiliki Pavlidou, Evangelia Papadimitriou, Mijomir Pelemis, Aantonis Antoniadis

**Affiliations:** * Aristotelian University of Thessaloniki, Thessaloniki, Greece; † Torlak Institute of Immunology and Virology, Belgrade, Yugoslavia; ‡ Institute for Tropical and Infectious Diseases, Belgrade, Yugoslavia

**Keywords:** Crimean-Congo hemorrhagic fever, virus isolation, PCR, Kosovo

## Abstract

*Crimean-Congo hemorrhagic fever virus* (C-CHFV) strains were isolated from a fatal case and the attending physician in Kosovo, Yugoslavia. Early, rapid diagnosis of the disease was achieved by reverse transcription-polymerase chain reaction. The physician was successfully treated with oral ribavirin. These cases yielded the first genetically studied C-CHFV human isolates in the Balkans.

Crimean-Congo hemorrhagic fever (C-CHF) is a tickborne hemorrhagic fever with documented person-to-person transmission and a case-fatality rate of approximately 30%. Nosocomial outbreaks caused by *Crimean-Congo hemorrhagic fever virus* (formal abbreviation, C-CHFV; genus *Nairovirus*, family *Bunyaviridae*) have been reported in several countries. This widespread virus has been found in Africa, Asia, the Middle East, and eastern Europe. C-CHFV was first identified in the Balkans in 1954. In 1973, Gligic et al. isolated three C-CHFV strains (Ciflik 1, 6, and 11) from two *Hyalomma* *plumbeum plumbeum* and one castor bean tick (*Ixodes ricinus*) (1). Since then, C-CHF has occurred every year in the Balkan Peninsula, with the number of cases related to the number of ticks infected. During the spring and summer of 2001, an outbreak of C-CHF occurred in Kosovo, with 69 suspected cases, 18 of them laboratory or clinically confirmed, 6 of them fatal (2).

## Case Reports

On June 30, 2001, a 43-year-old woman (the patient with the index case), living in Kosovska Mitrovica, Kosovo, became severely ill and was admitted to the hospital with high fever, headache, nausea, generalized myalgia, and disorientation. Two days later her condition rapidly deteriorated; bleeding from the **gastrointestinal tract, **she was transferred to the Institute for Tropical and Infectious Diseases in Belgrade, where she died a few hours after admission.

Blood cultures were negative for bacterial pathogens. C-CHF and hemorrhagic fever with renal syndrome (HFRS) were suspected, considering the clinical picture of the patient and the epidemiology of the diseases in this geographic area. A serum sample collected before death was tested for immunoglobulin (Ig) G and IgM specific for a variety of microorganisms (including hantaviruses, rickettsiae, and leptospira) by enzyme-linked immunosorbent assay and indirect immunofluorescent assay (IFA). No specific antibodies were detected.

To detect C-CHFV antibodies, the patient’s serum was tested in twofold dilutions (initial dilution 1:8) with fluorescein-labeled goat anti-human immunoglobulin (Gibco-BRL Diagnostics, Madison, WI) on spot slides containing Vero E6 cells (ATCC CRL 1586), with approximately 50% of the cells infected with the prototype Nigerian C-CHFV strain (IbAr 10200). Titers were recorded as the greatest dilution of serum at which characteristic cytoplasmic immunofluorescence is detected. No specific antibodies were detected.

On July 6, the 38-year-old physician who had intubated the index patient when she was admitted became severely ill, with high fever, asthenia, petechiae, **exanthema of soft palate, **pneumonia symptoms, leukopenia, and thrombocytopenia **(leukocyte count: 3x10^9^/L; erythrocyte count: 80x10^9^/L).** No evidence of disseminated intravascular coagulation was observed.

To detect antigen in the secondary patient’s serum, the immunoprecipitation procedure was used. Patient’s serum sample and sera from known C-CHF–positive patients, as well as hyperimmune human gamma globulin against C-CHFV, were placed in the wells in agar plates and left overnight to diffuse; precipitin lines were formed where the concentration of the antigen and antibodies was serologically equivalent (3).

We extracted viral RNA from serum and whole blood samples from both patients. Complementary DNA was amplified by nested polymerase chain reaction (PCR) by using primers from the small (S) RNA segment of C-CHFV (4). We found a specific 260-bp band in both cases. Nucleotide sequence analysis was performed by using the OpenGene automated DNA sequencing system (Visible Genetics Inc., Toronto, Canada). As expected, the two sequences were identical. We compared the resulting sequences with those available in the GenBank database using the BLAST tool (available from: URL: http://www.ncbi.nlm.nih.gov/BLAST/). We observed a similarity to C-CHFV S segment sequences.

Since strong evidence exists that ribavirin is effective for treating C-CHF (5,6), oral ribavirin was given to the second patient (800 mg three times on day 1, followed by 600 mg three times a day for the next 11 days). He responded successfully and, after 48 hours, became afebrile; his hematologic and biochemical parameters returned to normal. No side effects of ribavirin were present. On July 10, low titers (1:64) of IgM antibodies were detected, while on July 16, high titers (1:2,048) of IgG antibodies were detected in patient’s serum samples.

The patient was placed in isolation, and all personnel wore protective clothing, such as gowns, masks, and gloves. All contact persons in the hospital were informed and advised to contact a physician if any symptoms occurred. No additional persons became ill. Samples taken from the driver and the nurse who transferred the woman from the local hospital to Belgrade were tested by IFA for the presence of antibodies to C-CHFV and were found negative, even a month after the woman’s death.

Whole blood of both C-CHF cases (10% suspension in phosphate-buffered saline) was injected separately into 25-mL flasks containing Vero E6 cells. The cells were checked for C-CHFV antigen by immunofluorescence assay with hyperimmune human globulin against C-CHFV (strain Hodja). Positive cells were detected in both flasks on the 6th day after injection. The supernatant of the cells was used to infect fresh E6 cultures, and the virus was passaged six further times. We designated the virus from the fatal C-CHF case as C-CHFV/Kosovo/9553/2001, and the virus from the infected physician as C-CHFV/Kosovo/9717/2001.

Extraction of viral RNA of cell culture supernatants, amplification, and phylogenetic analysis showed that the obtained sequences were identical to each other and to the sequences amplified from the clinical samples. The nucleotide sequences were submitted to GenBank and assigned accession numbers AF428144 (for the index case) and AF428145 (for the secondary case). Phylogenetic analysis showed that these sequences clustered together with Drosdov strain (a C-CHFV strain isolated from blood of a patient in Russia), differing from it by 4%, while the genetic difference from the prototype C-CHFV Nigerian strain IbAr 10200 was 17% (Figure).

**Figure:**
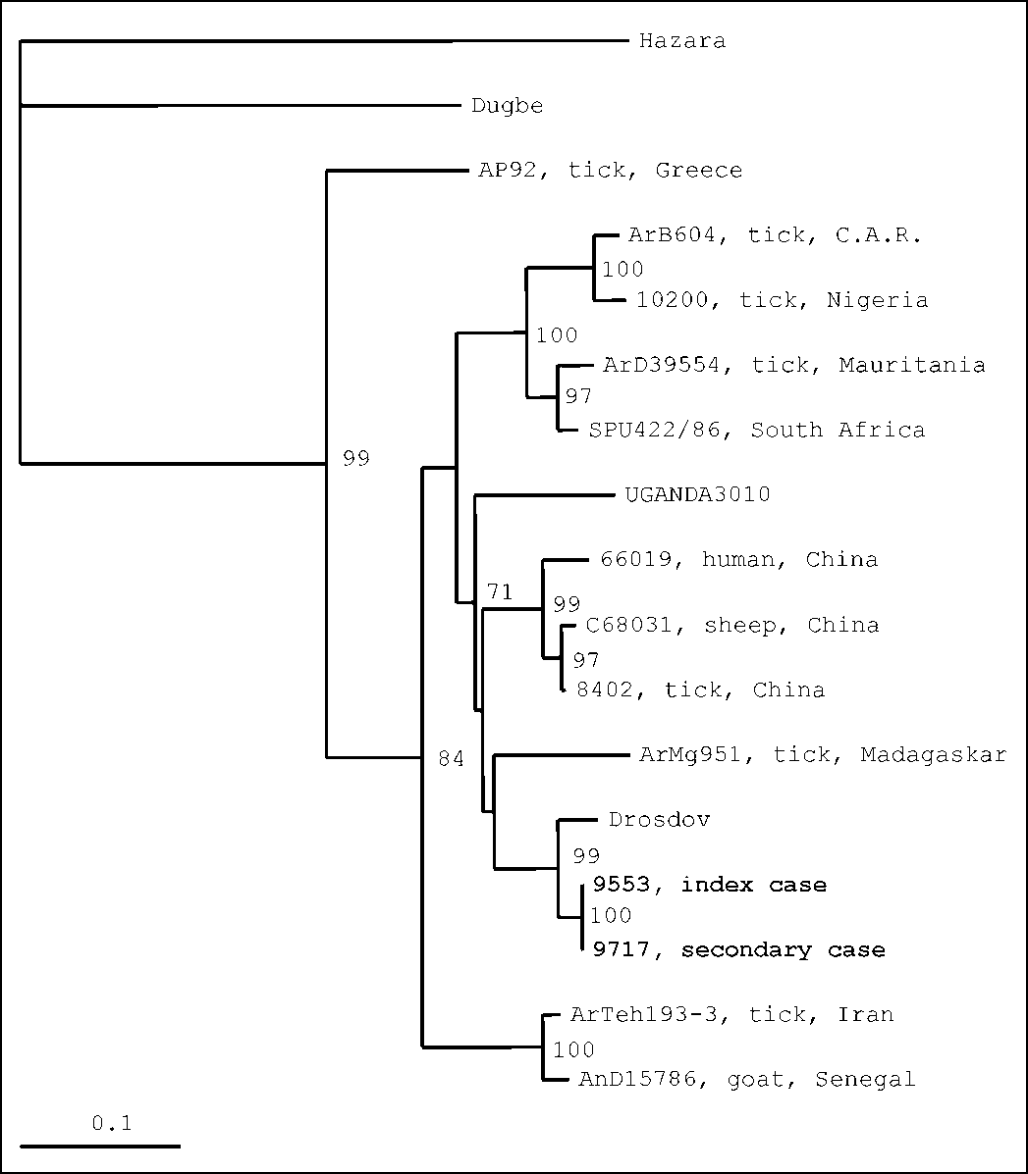
Phylogenetic relationships based on 255-nt fragment from the small RNA segment between sequences obtained from this study and respective representative Crimean-Congo hemorrhagic fever strains from GenBank. In the phylogenetic tree, sequences of other two nairoviruses, *Dugbe* and Hazara, were included; Hazara virus was used as outgroup. The numbers indicate percentage bootstrap replicates (of 100) calculated by using SEQBOOT, DNADIST, FITCH, and CONSENSE from the PHYLIP package (7); values <70% are not shown. Horizontal distances are proportional to the nucleotide differences. The scale bar indicates 10% nucleotide sequence divergence. Vertical distances are for clarity only.

## Discussion

Because of the severity of C-CHF, this illness has been difficult to study, either for diagnostic or therapeutic improvements. Antibodies to the virus are not present until 5 days after onset of illness. In addition, patients who have died of C-CHF do not usually develop a measurable antibody response. In these patients, diagnosis is achieved either by isolation of the virus from blood or tissue samples taken in the first days of illness or by molecular methods. In our first patient, who died, no antibodies were detected. Similarly, no antibodies were present during the first days of illness in the secondary case. Immunoprecipitation used for antigen detection gave the first evidence for C-CHF infection in the secondary case. However, reverse transcription (RT)-PCR established the diagnosis of the disease in both cases; sequencing the PCR amplicons confirmed the result. To our knowledge, this secondary case is the first in which PCR was used for rapid diagnosis, although use of PCR on stored acute-phase samples of infected patients has been described (4,8).

In addition, phylogenetic analysis was informative in comparing the virus strains at the genetic level, giving insights into the molecular epidemiology of the disease. The Greek C-CHFV strain (AP92), not yet associated with disease in humans, differs greatly from the Kosovo strains (24.3% nucleotide difference), forming an independent phylogenetic clade. Isolation of the virus, the standard for diagnosis, was achieved in both cases.

The first evidence of the in vitro activity of ribavirin against C-CHFV and its prophylactic use in patients’ contacts occurred in 1989 (9), and the effect of oral ribavirin on outcome in C-CHF patients was described in 1995 (6). Fisher-Hoch et al. stated that, because of the low frequency of this disease and its occurrence in rural, often remote, populations, a formal drug trial is unlikely. They suggested that the only likely way to gather sufficient information on therapy was through an accumulation of reports on similar clusters as they occur (6). Ribavarin is certainly more effective in intravenous form, rather than oral; however, intravenous ribavarin is difficult to obtain. For this reason, in treating the secondary case, we used its oral form. Recovery was observed a short time after initiation of treatment, without any side effects of ribavirin.

Although the risk for fatal outcome of the secondary case was not as high, based on criteria described by Swanepoel et al. (10), we concluded that ribavirin was effective in the patient, as he recovered rapidly after initiation of the treatment and had a successful immune response.

Concerning the origin of infection of the primary case, no details are available, such as a tick bite or any contact with C-CHF cases in the rest of Kosovo, where a C-CHF outbreak was taking place. No other C-CHF case had been reported from this region. The first patient died a few hours after admittance to the hospital, and the number of contact persons was limited. No one had any symptoms or C-CHF antibodies, except the physician who performed the intubation. He probably came in contact with patient’s excreta, despite protective measures.

No tertiary case was detected, as in C-CHF nosocomial outbreaks in other countries (Pakistan, Dubai, and South Africa) (6,11–13). The virulence of the virus is likely diminished on passage, and infectivity from secondary cases is unusual (12).

Further studies are needed both in diagnostic and treatment fields to elucidate this life-threatening disease. In addition, studies at the molecular level might relate the genetic variation with the pathogenicity of the strains, rendering feasible the production of an effective vaccine.
